# Design and Characterization of Citronella Oil-Loaded Micro-Emulgel for the Treatment of *Candida Albicans* Infection

**DOI:** 10.3390/gels9100799

**Published:** 2023-10-05

**Authors:** Shambhvi Singh, Shikha Baghel Chauhan, Charu Gupta, Indu Singh, Annie Gupta, Shwetakshi Sharma, S. M. Kawish, Shakilur Rahman, Muzaffar Iqbal

**Affiliations:** 1 Amity Institute of Pharmacy, Amity University, Noida, Uttar Pradesh 201301, India; shambhvi5282@gmail.com (S.S.);; 2 Amity Institute of Herbal Research & Studies, Amity University Noida, Noida, Uttar Pradesh 201313, India; cgupta@amity.edu; 3 Department of Pharmaceutics, School of Pharmaceutical Education and Research, Jamia Hamdard University, New Delhi, Delhi 110062, India; 4 Department of Anesthesiology and Perioperative Medicine, University of Alabama at Birmingham, Birmingham, AL 35249-6810, USA; 5 Department of Pharmaceutical Chemistry, College of Pharmacy, King Saud University, Riyadh 11451, Saudi Arabia

**Keywords:** micro-emulgel, citronella oil, cinnamon oil, *Candida albicans*, microemulsion

## Abstract

The purpose of the current study was to prepare and evaluate a citronella oil-loaded microemulsion-based micro-emulgel for the treatment of *Candida albicans*. The primary objective was to use the skin to transfer hydrophobic medications into the bloodstream. The formulation included cinnamon oil as an antifungal oil and citronella oil as an active pharmaceutical ingredient, respectively. Tween 80 and PEG 200 were used as the surfactant and co-surfactant, respectively, to create phase diagrams. Carbopol 940, one of the frequently used polymers, was investigated for its ability to prepare gel formulations. The optimized (F3) batch contained the highest percentage (87.05 ± 0.03%) of drug content and, according to the statistics provided, had the highest drug release rate of around 87.05% within 4 h. The Korsmeyer–Peppas model with *n* value of 0.82, which is in the range 0.5–1, had the highest r^2^ value, indicating that release following non-Fickian/anomalous diffusion provided a better dimension for all of the formulations. The optimized (F3) formulation had stronger antifungal activity in comparison to other formulations. This leads to the conclusion that citronella oil can be made into a micro-emulgel, which may improve its release in aqueous systems while maintaining a high level of drug release at the target site.

## 1. Introduction

Skin is an essential feature of the human body which provides a shield from external foreign particles [1]. Skin, on the other hand, is much more susceptible to bacterial infection [2]. Skin illnesses such as eczema, cellulitis, and antibacterial infections caused by bacteria such as *Streptococci*, *Staphylococci*, *Escherichia coli*, and others have become common in today’s world [3,4]. Fungi are another type of invading organism that can cause serious fungal infections such as vaginal and superficial candidiasis, ringworm, and athlete’s foot [5]. The most common forms of fungal skin infections are caused by fungi that flourish in moist, humid settings. *Candida albicans*, *Candida glabrata*, *Candida krusei*, *Trichophyton rubrum*, *Tinea capitis*, *Tinea barbae*, *Malassezia furfur*, and *Cryptococcus neoformans* are only a few of the fungi that cause such illnesses [6,7]. Topical dosage forms such as creams, gels, sprays, and lotions are widely available and contain synthetic active pharmaceutical ingredients (APIs) such as triazole derivatives (fluconazole, eberconazole, ketoconazole, and luliconazole); in addition, there are oral antifungal medications such as tablets, capsules, and suspensions containing fluconazole, itraconazole, and micafungin [8]. However, due to the progressive emergence of fungal resistance and its side effects, as well as treatment costs, the use of conventional antibiotics now shows major limitations for antifungal treatment. On the other hand, essential oils, apart from having lower toxicity and better biodegradability, are eco-friendly in nature as compared with conventional antibiotics [9,10,11].

**Figure 1:**
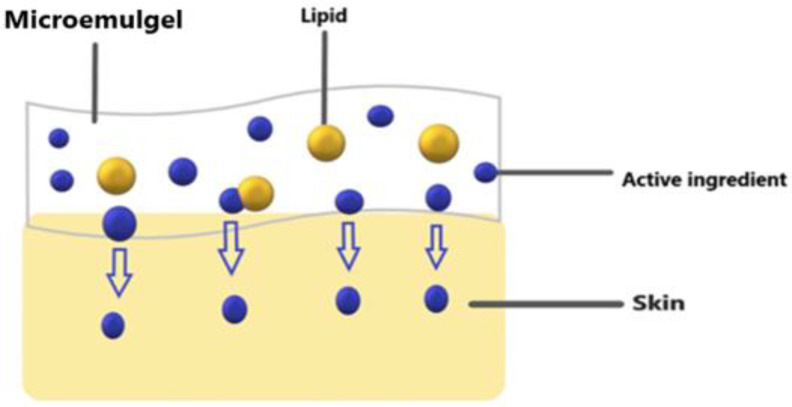
Showing micro-emulgel pathway on skin.

Micro-emulgels, being a combination of microemulsion and gel, are a unique and novel dosage form that can cope with the problems associated with conventional dosage forms and have advantages in gel and microemulsion form. Compared to cream, micro-emulgels have more penetration power in skin. Being a dual mechanism using an emulsion and gel, micro-emulgels (Figure 1) are regarded as one of the most promising technologies among innovative drug delivery systems and have emerged as a novel approach for topical delivery of drugs [12]. Additionally, adding gel to an emulsion boosted its stability [13]. The outstanding solubilizing and skin penetration properties of the microemulsion technology were the deciding factors during its formulation development [14]. The primary requirement for the formulation of an emulgel is the appropriate selection of the oil phase, emulsifier, and gelling agent Therefore, oils, surfactants, and co-surfactants must be carefully screened and optimized during the preparation of micro-emulgels. The choice of these is made based on the solubility profile of the API. The API penetrates the skin more deeply when there is oil present. A large interfacial area is created by the creation of tiny droplets in the microemulsion, which increases the surface area available for medication absorption [15].

Candidiasis is the most prevalent fungal disease, including a wide range of infections from superficial to systemic with high mortality rates. *Candida albicans* is the most studied species for candidiasis infection and is becoming resistant towards existing antifungal drugs. It is a diploid yeast, which means it has two sets of eight chromosomes. Its genome is around 16 megabytes in size and has a total of 6159 coding genes. It is a frequent cause of invasive fungal infections and is also considered as one of the main agents responsible for opportunistic pathogenic infections [16,17]. Citronella and cinnamon oils are essential plant oils obtained from *Cymbopogon winterianus* and *Cinnamon cassia*, respectively [18]. Citronella is a monoterpenoid that is the main component of citronella oil and gives it its distinct lemon aroma. It is a monoterpene as well as an aldehyde and acts as an antifungal agent both single as well as with its metabolite. Citronella oil is water-soluble, ethanol-soluble, and glycerol-insoluble. Cinnamon is a common spice that has been used for thousands of years by various cultures all over the world [19]. It is derived from several components of a tropical evergreen tree of the genus Cinnamomum. Several in vivo and in vitro investigations have revealed that cinnamon essential oils and their primary constituents have significant inhibitory effects against a variety of fungi, including *Coriolus versicolor*, *Laetiporus sulphureus*, *Eurotium* spp., *Aspergillus* spp., and *Penicillium* spp. [20,21,22,23]. Fungicidal and inhibitory efficacy of citronella and cinnamon oils on Candida albicans biofilms has already been reported in the literature [24,25]. Recently, it was reported that the antimicrobial and anticancer properties of citronella oil were promisingly improved once it was used as a nanoemulsion form of formulation [26]. Therefore, the current study proposes to develop and analyze a citronella oil-loaded microemulsion-based gel to enhance its solubility and permeability and for improved antifungal efficacy by using carbopol 940 as the gel foundation.

## 2. Results and Discussion

### 2.1. Preformulation Study of Citronella Oil

#### 2.1.1. Physical Appearance Test

The physical appearance of the citronella oil is mentioned in Table 1. It was in liquid form and was slightly yellow in color, having intense lemon rose odor.

#### 2.1.2. Determination of Lambda Max of Citronella Oil

**Figure 2:**
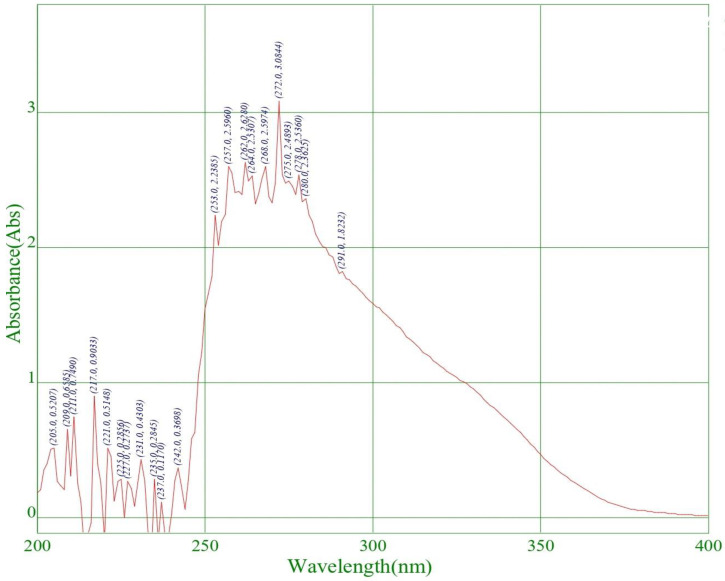
Determination of λmax in DMSO solution.

For the quantitative analysis of citronella oil, its λmax was measured initially by a UV spectrophotometer in dimethyl sulphoxide (DMSO). The scanning of the drug (citronella oil) was carried out in a range of 200–900 nm, and its λmax was found to be 272 nm in DMSO, which is highlighted below in Figure 2.

#### 2.1.3. Calibration Curve of Citronella Oil

**Figure 3:**
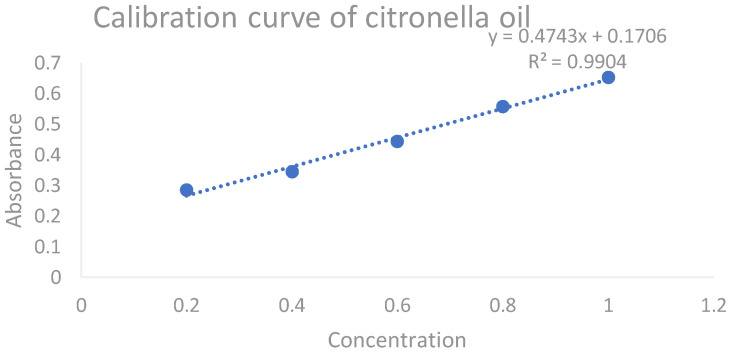
The calibration curve of citronella oil.

An amount of 1 mL of citronella oil was properly measured with a measuring cylinder and diluted in 99 mL of DMSO to make a 100 mL stock solution. Appropriate aliquots of 0.2, 0.4, 0.6, 0.8, and 1 µL were transferred to a series of 10 mL volumetric flasks, the volume was built up to 10 mL with DMSO, and the absorbance of these solutions was measured at a predetermined maximum absorbance (272 nm). The procedure was completed in triplet sequence. The obtained average slope was 0.4743× + 0.1706 with an r^2^ value of 0.9904. By using this calibration curve, the concentration of citronella oil in different CITRO-microemulgel formulations was measured. The representative calibration curve of citronella oil in DMSO is presented in Figure 3.

#### 2.1.4. Phase Study

**Figure 4:**
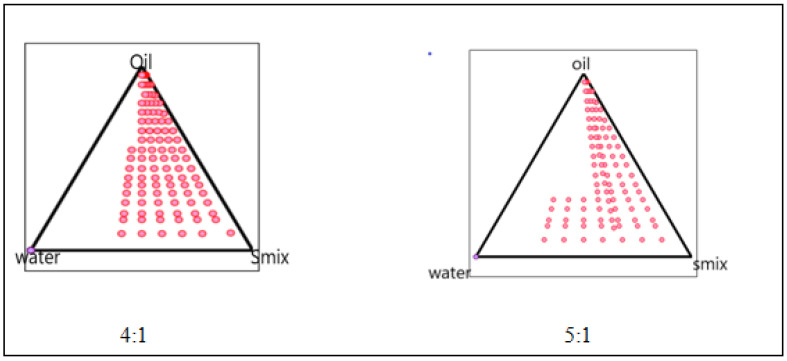
Pseudo-ternary phase diagram of 4:1 5:1.

The phase diagram was created using the water titration method to establish the proper components and their concentration range. The co-surfactant was chosen as PEG 200, the surfactant was chosen as Tween 80, and the oil phase was chosen as citronella oil. Distilled water was used in the oil phase. The entire amount of surfactant, lipid, and water in the overall combination was stated as a percentage of the total amount of water. The (S mix) of surfactant and co-surfactant was mixed in various weight ratios (4:1 5:1) to identify the ideal ratio that resulted in the biggest microemulsion area. For each phase diagram, oil and Smix were combined in varying ratios of 1:9 to 9:1 in different vials. The oil-to-surfactant ratios were 1:9, 2:8, 3:7, 4:6, 5:5, 6:4, 7:3, 8:3, 8:2, and 9:1. Following that, the mixture was titrated with water. Visual observations for transparent and easily flowable (*o*/*w*) microemulsion were made after the mixture was stirred for 2–3 min and equilibrated for 30 min. On a pseudo-ternary phase diagram, the physical condition of microemulsion was depicted, with one axis representing Smix, the other water, and the third oil, as presented in Figure 4.

### 2.2. CITRO-Microemulgel Preparation

**Figure 5:**
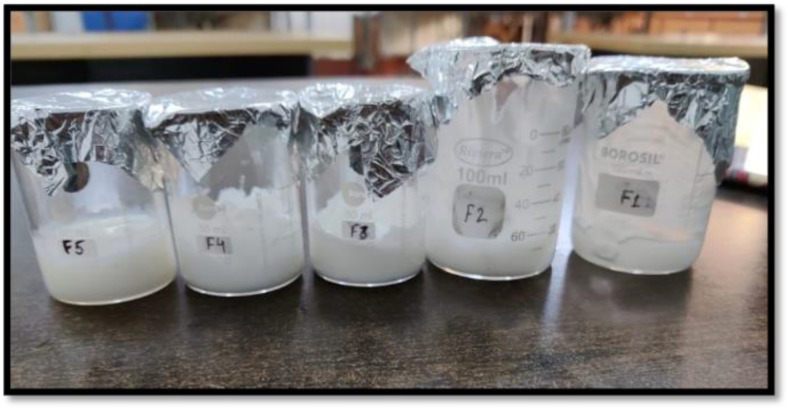
CITRO-microemulgel (F1–F5).

A total of five formulations of CITRO-microemulgel were prepared by using citronella oil as an API with different concentrations of 2, 4, 5, 3, and 1%, whereas cinnamon oil was used as an antifungal oil and penetration enhancer (Table 2). Carbopol 940 (1–2%) was used as a gelling agent. Tween 80 and PEG 200 were used as Smix in the formulation. TEA was used as a pH adjuster to make the final formulation pH 6–6.5, which was considered to avoid the risk of any irritation. The prepared five different formulations of CITRO-microemulgel (F1–F5) are presented in Figure 5.

### 2.3. Evaluation Parameters of CITRO-Microemulgel

#### 2.3.1. Organoleptic Properties and Appearance

The results for the organoleptic properties and appearance of formulations F1–F5 are presented in the below Table 3. No phase separation and grittiness was seen in all F1–F5 formulations of CITRO-microemulgel. It was observed that the formulation F3 showed the best organoleptic properties in terms of clarity, color, consistency, and phase separation among the five formulations. The consistency of the F3 formulation was more flowable in nature in comparison to other formulations.

#### 2.3.2. pH Measurement

The pH value of the micro-emulgel formulation should be compatible with skin pH, thereby avoiding the risk of skin irritation during application. TEA is commonly used for pH adjustment in emulgel formulations, considering carbopol is widely used as a thickener and among other things. TEA is necessary to uncoil the polymer chain of carbopol and for the formation of a gel structure. The measured pH values for all five formulations (F1–F5) are presented in Table 4. All of the formulations F1–F5 had a pH value between 5.6 and 6.5, which is regarded as appropriate to prevent any danger of application-related discomfort and is suitable for use on human skin. Among them, the F3 formulation has the best pH, 6.5, among other formulations, which is considered to avoid the risk of any irritation to skin during its application.

#### 2.3.3. Spreadability

**Figure 6:**
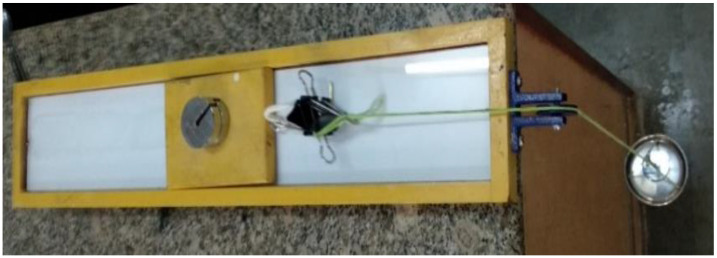
Showing spreadability of CITRO-microemulgel.

The spreadability is one of the most important properties of topical formulations that affect therapeutic efficacy and patient compliance. It is an important criterion for inspecting uniformity, ease, and application of transdermal formulations. For the formulations F1 to F5, the spreadability of CITRO-microemulgel ranged from 33.74 g.cm/min to 68.74 g.cm/min, respectively. The F3 formulation has the best spreadability (68.74/753 g.cm/min) compared to all remaining formulations. The spreadability of CITRO-microemulgel formulations from F1 to F5 is shown in Table 5 and Figure 6 below.

#### 2.3.4. Drug Content

Determination of drug content is necessary to evaluate the uniform distribution of the drug in the formulation and to ensure a minimum batch-to-batch variation. The content in the micro-emulgel was determined by dissolving the known weight of the CITRO-microemulgel formulation in 10 mL of ethanol to prepare appropriate dilutions. The solution was stirred for 3–4 h on a magnetic stirrer to ensure complete dissolution of the formulation in ethanol. Later, the resulting solution was filtered through Whitman filter paper to provide a clear solution. This procedure was used to determine the drug concentration of the emulgel. A UV spectrophotometer was used to test the absorbance of the citronella oil at 272 nm, respectively. The drug concentration of citronella oil in CITRO-microemulgel formulations was calculated using the simultaneous equation. The drug content of citronella oil is presented in Table 6, and it was found to be in the range of 77.09 to 87.05 in all five (F1–F5) formulations. The F3 batch contains the highest percentage of drug content (87.05%). These results confirmed that the drug was evenly dispersed throughout the CITRO-microemulgel, according to the analysis of the drug content.

#### 2.3.5. In Vitro Drug Release

**Figure 7:**
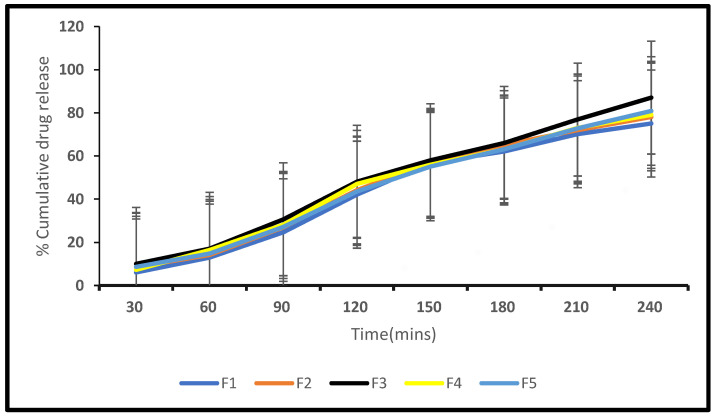
In vitro release profile of F1–F5 formulations.

Different CITRO-microemulgel formulations were tested in vitro for drug release using a dialysis membrane and are shown and plotted in Table 7 below. According to the statistics provided, formulation F3 had the highest drug release rate of around 87.05 percent within 4 h, as shown in Figure 7.

#### 2.3.6. Drug Release Kinetics

The drug release profile was also assessed for “fit” into various mathematical model equations such as zero order, first order, Korsmeyer–Peppas, and Higuchi matrix. These kinetic models were utilized to better understand the drug release mechanism of the CITRO-microemulgel. The model equation’s r^2^ value is provided in Table 8. The model with the highest r^2^ value was chosen as the best-fit model for the formulation. The Korsmeyer–Peppas model with *n* = 0.82, which is between 0.5–1, had the highest r^2^ value, indicating that release followed non-Fickian/anomalous diffusion and providing a better dimension for all of the formulations.

#### 2.3.7. Microbial Assay

**Figure 8:**
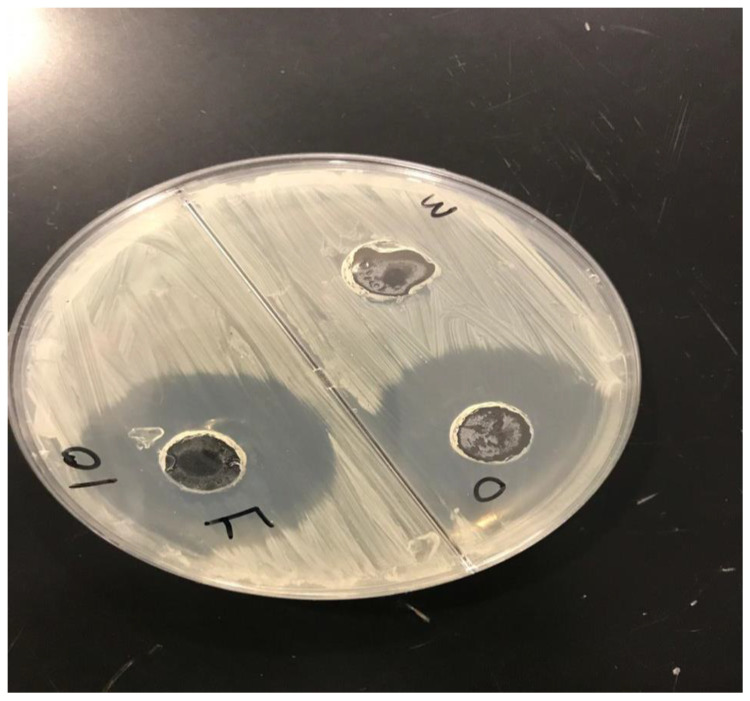
Zone of inhibitions of optimized CITRO-microemulgel F3 (F) in comparison to other formulation (O) and control (M).

Antifungal activity tests were carried out using the good diffusion method, which involved assessing the zone of inhibition (in mm). The gel’s results revealed antifungal effectiveness of different formulations against fungus-causing *Candida albicans*. Figure 8 depicts a study of the zone of inhibition for the formulation’s antifungal efficacy. The formed gel F3 (F) had stronger antifungal activity than the other formulation (O) and the control with a zone of inhibition diameter of 35 mm for *Candida albicans*. As a result, this topical formulation (F3) is considered to be suitable for the treatment of fungal infections.

## 3. Conclusions

In this study, a total of five formulations of CITRO-microemulgel were prepared by using citronella oil as an active pharmaceutical ingredient with different concentrations of 2, 4, 5, 3, and 1% (*w*/*v*), whereas cinnamon oil was used as an antifungal oil and penetration enhancer. Among the five formulations, F3 showed the best organoleptic properties in terms of clarity, color, consistency, and phase separation. F3 showed the most favorable pH (6.5 ± 0.12) and contained the highest percentage of drug content, which was 87.05 ± 0.03%. According to the statistics provided, formulation F3 of CITRO-microemulgel had the highest drug release rate of around 87.05% within 4 h. The model with the highest r^2^ value was chosen as the best-fit model for the formulation. The Korsmeyer– Peppas model with n=0.82, which is between 0.5–1, had the highest r^2^ value, indicating that release followed non-Fickian/anomalous diffusion and providing a better dimension for all of the formulations. The optimized CITRO-microemulgel (F3) showed a highest zone of inhibition diameter of 35 mm for *Candida albicans*, which confirms that this can be a promising agent for eradicating infection.

## 4. Material and Methods

The citronella oil and cinnamon oil were purchased from Allin exporters (Noida, Uttar Pradesh, India). Tween 80, carbopol 940, and methyl paraben were obtained from CDH Fine Chemicals (New Delhi, India). The PEG-200 was from Seva Fine Chemicals (Ahmedabad, India), while the triethanolamine (TEA) was from Fisher Scientific (Loughborough, United Kingdom). Highly pure deionized water was used for aqueous solution preparation during formulation development analysis. All other organic solvents and chemicals used for formulation development and analysis were of analytical and chromatographic grade.

### 4.1. Preparation of Citronella Oil-Loaded Microemulsion

By using the phase titration method, the microemulsion of citronella oil was prepared using tween 80 as a surfactant and PEG 200 as a co-surfactant, respectively. The different combinations of surfactant and co-surfactant in different ratios referred to as Smix ratios, oil, and water were used to investigate the phase behavior by using a simple titration method. Oil-to-Smix ratios of 1:1, 1:2, 1:3, 1:4, 4:1, and 5:1 were used to create a total of 6 ternary phase diagrams. To create a pseudo-ternary phase diagram for each Smix ratio, solutions comprising oil and Smix were made with volume ratios ranging from 1:9 to 9:1 and titrated drop by drop with water (kept in continuous stirring) at room temperature until turbidity appeared.

### 4.2. Preparation of CITRO-Microemulgel

**Figure 9:**
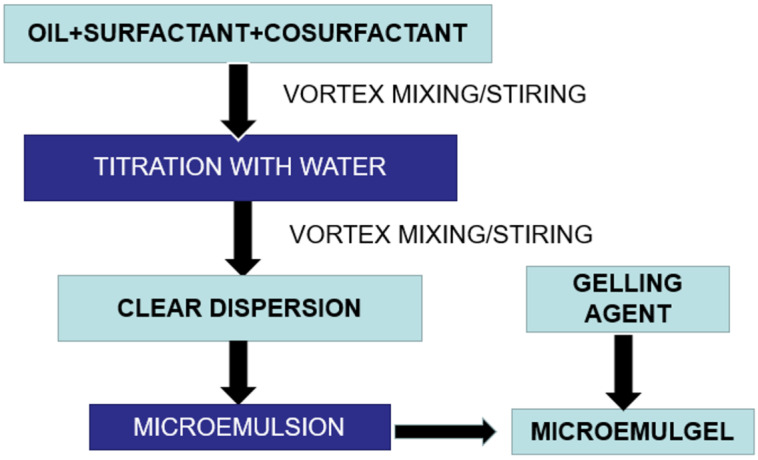
Flow chart for preparation of CITRO-microemulgel.

The gelling agents impact the consistency of dosage form, spreading coefficient, viscosity of the formulation, drug release from the formulation, and stability of a system. Generally, gelling agents are used to increase the consistency of any formulation. The gel phase was made by dispersing carbopol 940 (a gelling agent) in water with the use of a mechanical stirrer at 1000 rpm until no lumps remained. The cinnamon oil was added as an antifungal oil and penetration enhancer. After that, drop by drop, TEA was added to adjust the pH to 6–6.5. Finally, using a mechanical stirrer set to 1000 rpm for 15 min, the produced emulsion was disseminated in the gel system in a 1:1 ratio [27]. The schematic flow chart for preparation of CITRO-microemulgel is presented in Figure 9.

### 4.3. Evaluation Parameters

#### 4.3.1. Organoleptic Properties and Physical Appearance

The prepared CITRO-microemulgel was kept for 24 h at different specified temperature conditions, i.e., 8 °C, 25 °C, and 40 °C with 75% relative humidity (RH), and was visually evaluated for assessment of their organoleptic (color and odor) and physical stability parameters (grittiness, clarity, phase separation, and consistency) [28].

#### 4.3.2. pH

The pH values of 1% aqueous solutions of the prepared gellified emulsion were measured by a digital pH meter. The prepared one gram of CITRO-microemulgel was dissolved in 25 mL of distilled water, and the electrode was immersed in the solution for 30 min until a steady reading was obtained. Each formulation’s pH was measured three times, and the average values were calculated [29].

#### 4.3.3. Spreadability

The distribution of the CITRO-microemulgel formulation was examined after 48 h of preparation. A petri dish with 1 g of emulgel was placed inside a 1 cm diameter circle pre-marked on a glass plate, followed by a 75 g glass plate. On a glass plate, the weights were then left to rest for 5 min. The spread CITRO-microemulgel resulted in an increase in diameter, which was noticed. It was calculated by the formula [30]:

*S* = *m·l*\*t* (1)

where *S* = Spreadability

*m* = Weight of an upper glass plate and weight put on it (g)

*l* = Diameter of spreading emulgel (cm)

*t* = Time taken to spread emulgel (min) 

#### 4.3.4. Drug Content Determination

One gram of CITRO-microemulgel was dissolved in a suitable solvent and filtered to obtain a clear solution. Its absorbance was determined by using a UV spectrophotometer. A conventional drug plot was made in the same solvent. Concentration and drug content were determined by plotting absorbance values on the same standard plot [31].

*Drug Content* = (*Concentration* × *Dilution Factor* × *Volume taken*) × *Conversion Factor* (2)

### 4.4. In Vitro Drug Release

The CITRO-microemulgel in vitro diffusion was studied using a dialysis membrane. The membrane was carefully clamped to one end of the dialysis cell’s hollow glass tube after being soaked in phosphate buffer, pH 7.4 (PB) for 6–8 h (2.3 cm diameter; 4.16 cm area). A beaker containing 250 mL of phosphate buffer was employed as the receptor compartment. The membrane was then evenly coated with 1 g of CITRO-microemulgel. The donor compartment remained in contact with the receptor compartment, and the temperature was fixed at 37.5 °C. Externally driven Teflon-coated magnetic bars agitated the liquids on the receptor side. An amount of 5 mL of solution was pipetted out from the receptor compartment at predefined time intervals and immediately replaced with fresh 5 mL phosphate buffer. The drug concentration in the receptor fluid was measured spectrophotometrically in comparison to an acceptable blank. The experiment was conducted three times [32].

### 4.5. Microbial Assay

Trench plates were used as a method. They are a technique for figuring out whether a substance has bacteriostatic or fungistatic properties and are mostly utilized in formulations for semisolids. The previously produced agar-dried plates of Sabouraud were used. Three grams of gellified emulsion were placed in a trench that had been carved in the plate. Freshly made culture loops were smeared across the agar at a straight angle from the ditch to the edge of the plate. Following 18 to 24 h of incubation at 25 °C, the fungal growth was checked, and the percentage of inhibition was estimated as follows [33].

% *inhibition = L*2*\L*1 × 100 (3)

where *L*1 = Total length of the streaked; *L*2 = Length of Inhibition.

## Figures and Tables

**Table 1:** Observation table of physical appearance of drug.

Evaluation Parameters	Observations
Color	Slightly yellow
Physical state	Liquid
Odor	Intense lemon-citronella-rose odor

**Table 2:** Formulation table for CITRO-microemulgel (F1–F5).

Material	F1	F2	F3	F4	F5
Citronella oil (mL)	2%	4%	5%	3%	1%
Carbopol 940	1	0.7	0.5	1.5	2
Tween 80	5	5	5	5	5
PEG 200	1	1	1	1	1
Cinnamon oil	2	2	2	2	2
Triethanolamine	1	1	1	1	1
Methyl paraben	0.03	0.03	0.03	0.03	0.03
Distilled water	100	100	100	100	100

**Table 3:** Physical appearance and phase separation.

Formulation	Grittiness	Clarity	Phase Separation	Color	Consistency
F1	no	+	no	W	gel
F2	no	++	no	OW	gel
F3	no	++	no	W	flowable
F4	no	+	no	W	gel
F5	no	+++	no	W	less flowable

Clarity —+ (satisfied); ++ (good); +++ (excellent); W—white in color; OW—off-white.

**Table 4:** pH of prepared formulations.

Formulation Code	pH (Mean ± SD, *n* = 3)
F1	6.2 ± 0.1
F2	5.6 ± 0.24
F3	6.5 ± 0.12
F4	6.1 ± 0.22
F4	5.9 ± 0.10

**Table 5:** Showing spreadability of different formulations (F1–F5).

Formulation Code	Spreadability
F1	36.74/7.065 g.cm/min
F2	59.74/9.08 g.cm/min
F3	68.74/7.53 g.cm/min
F4	33.74/8.04 g.cm/min
F5	61.34/3.79 g.cm/min

**Table 6:** Drug content of CITRO-microemulgel (F1–F5 formulations).

Formulation	% Drug Content (Mean ± SD, *n* = 3)
F1	77.09 ± 0.01
F2	81.76 ± 0.04
F3	87.05 ± 0.03
F4	79.08 ± 0.07
F5	80.87 ± 0.02

**Table 7:** In vitro drug release of formulations (F1–F5).

Time (min)	F1	F2	F3	F4	F5
0	0	0	0	0	0
30	6.05 ± 0.02	8.54 ± 0.12	10.03 ± 0.14	7.25 ± 0.14	8.62 ± 0.04
60	12.9 ± 0.13	14.09 ± 0.22	17.03 ± 0.21	16.45 ± 0.15	14.72 ± 0.28
90	24.67 ± 0.22	26.89 ± 0.03	30.69 ± 0.20	28.06 ± 0.12	27.05 ± 0.32
120	42.07 ± 0.04	44.24 ± 0.04	48.07 ± 0.12	47.09 ± 0.23	43.67 ± 0.08
150	56.67 ± 0.11	57.01 ± 0.12	58.02 ± 0.06	55.87 ± 0.09	55.09 ± 0.42
180	62.08 ± 0.13	65.29 ± 0.23	66.09 ± 0.16	63.08 ± 0.18	63.07 ± 0.01
210	70.08 ± 0.14	72.05 ± 0.30	76.87 ± 0.14	73.08 ± 0.20	72.89 ± 0.61
240	75.06 ± 0.32	78.09 ± 0.13	87.05 ± 0.12	79.08 ± 0.14	80.87 ± 0.23

**Table 8:** Various kinetic models of different formulations.

Formulation	Zero Order r^2^	First Order	Higuchi	Korsmeyer	N
F1	0.9837	0.8204	0.8797	0.9409	0.77
F2	0.9818	0.787	0.8944	0.9653	0.74
F3	0.993	0.9033	0.9835	0.9794	0.82
F4	0.9857	0.782	0.9042	0.9638	0.78
F5	0.990	0.7888	0.894	0.9688	0.79

## Data Availability

Available upon request.
